# Dietary supplement use in elementary school children: a Japanese web-based survey

**DOI:** 10.1186/s12199-021-00985-7

**Published:** 2021-06-05

**Authors:** Kazue Ishitsuka, Satoshi Sasaki, Hidetoshi Mezawa, Mizuho Konishi, Maki Igarashi, Kiwako Yamamoto-Hanada, Shoji F. Nakayama, Yukihiro Ohya

**Affiliations:** 1 Medical Support Center for the Japan Environment and Children’s Study, National Center for Child Health and Development, 2-10-1 Okura, Setagaya-ku, Tokyo, 157-8535 Japan; 2 Department of Social and Preventive Epidemiology, Graduate School of Medicine, The University of Tokyo, 7-3-1 Hongo, Bunkyo-ku, Tokyo, 113-8654 Japan; 3 Department of Social and Preventive Epidemiology, School of Public Health, The University of Tokyo, 7-3-1 Hongo, Bunkyo-ku, Tokyo, 113-8654 Japan; 4 Department of Molecular Endocrinology, National Research Institute for Child Health and Development, 2-10-1 Okura, Setagaya-ku, Tokyo, 157-8535 Japan; 5 Centre for Health and Environmental Risk Research, National Institute for Environmental Studies, 16-2 Onogawa, Tsukuba, 305-8506 Japan

**Keywords:** Dietary supplement, Children, Socio-economic status, Sports participation

## Abstract

**Background:**

A variety of dietary supplements are commercially available. However, the efficacy and safety of dietary supplement use in children are not well established. Understanding dietary supplement use is important for developing public health policy regarding dietary supplements. This study aimed to investigate the types of dietary supplements used and characteristics of dietary supplement users among Japanese elementary school children.

**Method:**

We conducted a cross-sectional web-based questionnaire study. Dietary supplement use, socio-demographics, and health-related behaviors were assessed through mother-reported questionnaire. Types of dietary supplements were identified based on ingredient using product barcodes and brand names. Multivariate logistic regression analysis was conducted to investigate the socio-demographics and health-related behaviors associated with supplement use.

**Results:**

Among 4933 children, 333 (6.8%) were identified as dietary supplement users. The most common supplement was amino acids or protein (1.4%), followed by n–3 fatty acids or fish oil (1.0%), probiotics (1.0%), multivitamins (0.9%), multivitamin-minerals (0.8%), and botanicals (0.8%). Overall, any dietary supplement use was significantly associated with the highest frequency of sports participation (odds ratio [OR], 2.58; 95% confidence interval [CI], 1.65–4.02), highest household income (OR, 1.87; 95% CI, 1.13–3.10), highest maternal educational level (OR, 1.82; 95% CI, 1.31–2.52), and male sex (OR, 1.38; 95% CI, 1.09–1.75). The highest frequency of sports participation was significantly associated with higher odds of use of amino acids or protein (OR, 6.06; 95% CI, 1.78–20.6) and multivitamins (OR, 3.56; 95% CI, 1.11–11.5), compared to the lowest frequency of sports participation.

**Conclusion:**

This study showed that Japanese children primarily use non-vitamin, non-mineral supplements. Non-vitamin, non-mineral supplements should thus be included in future studies aimed at monitoring dietary supplement use. We also found that dietary supplement use in children was associated with sports participation. Guidelines for dietary supplement use for children, in particular sport participants, are needed.

**Supplementary Information:**

The online version contains supplementary material available at 10.1186/s12199-021-00985-7.

## Introduction

Nutrient intake in children and adolescents can influence growth and the risk of non-communicable diseases when they become adults [1]. Peak bone mass during adolescence is associated with osteoporosis in later life [2]. Research suggests that adequate intake of calcium and vitamin D during childhood and adolescence is associated with higher peak bone mass [2]. Adequate calcium and vitamin D intake during childhood may have preventive effect of future osteoporosis. Dietary guidelines recommend that nutrients be primarily consumed from foods [3, 4]. Several studies have shown, however, that intake of some nutrients including calcium, iron, and vitamin A from food is insufficient to meet the respective Dietary Reference Intake in a substantial number of children from a certain population [5, 6]. Dietary supplements may be a choice to fill this gap. Although generally safe, dietary supplement use can result in excessive intake of some vitamins and minerals, including vitamin A and iron [7].

Non-vitamin, non-mineral (NVNM) supplements are often used for health promotion and other purposes in addition to compensating for nutrient inadequacy from food [8]. For some NVNM supplements, toxicity may be an issue, including liver or renal toxicity [9, 10]. A case report suggested that Chlorella may be an allergen that causes tubulointerstitial kidney disease in children [9, 11]. Another case report suggested that noni may cause liver injury among children [10, 12]. The anthraquinones contained in noni are thought to produce oxygen-derived free radicals and cause oxidative stress, resulting in liver injury [12]. Understanding the current status and issues related to dietary supplement use is thus important for developing public health policy regarding dietary supplements.

Dietary supplements are defined as foods consumed orally as a capsule, tablet, powder, liquid, or other form to supplement the diet [13, 14]. Types of dietary supplements include (i) vitamins, (ii) minerals, (iii) herbs or other botanicals, (iv) amino acids, and (v) ingredients of a concentrate, metabolite, constituent, or extract [13, 14]. Vitamin or mineral (VM) supplements are major dietary supplements [15, 16]. Some studies have shown that users of VM supplements are more likely to have higher socio-economic status and greater sports participation than non-users [17–19]. While earlier studies have investigated VM use, few studies have examined use of NVNM supplements [17, 20]. A recent national survey in the USA showed that an increasing number of children and adolescents are using NVNM supplements, including amino acids, n–3 fatty acids, and botanical supplements [15].

We investigated the types of dietary supplements used and the characteristics of dietary supplement users among elementary school children in Japan. We used a questionnaire to inquire about the brands and manufacturers of supplements being used to investigate newly sold supplements and supplements marketed to children.

## Methods

### Study design and study participants

We conducted a cross-sectional study to collect information on dietary supplement use among elementary school children. The eligibility criterion for this study was elementary school children (grade 1–6, corresponding to 6–12 years old). Mothers of elementary school children registered with a research agency were sent an email inviting them to participate in this study and asking them to provide answers to a web-based questionnaire. The research agency, named Q life, provides access to study participants for research, along with access to websites with health and medical information. More than five million individuals are registered with Q-life and their collaborate agencies. Recruitment was continued until the number of study participants reached the target (over 4000). This study was conducted from September 22 to October 31, 2017.

**Fig. 1:**
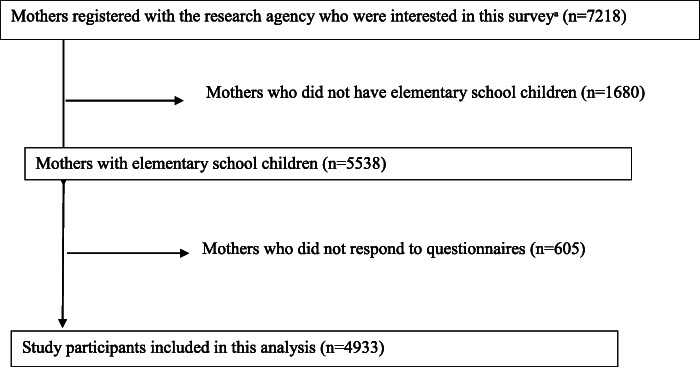
Flow diagram of study participants. Superscript small letter “a” indicates that mothers were recruited through e-mail and the website of a research agency

Figure 1 shows a flow diagram of the study participants’ selection. Of the 5538 mothers eligible for this study, 4933 completed the questionnaire on dietary supplement use. Duplication of study participants was checked using the mother’s internet cookies, IP address, ID number, and email address. The study protocol was reviewed and approved by the institutional review board of the National Center for Child Health and Development (Number 1440). Electronic informed consent was obtained from all mothers.

The participants lived throughout all 47 prefectures in Japan. Distribution of prefectures in which the children lived, household income, and maternal age were comparable to those in the national survey [21–23]. Furthermore, distribution of the children’s height and weight in the present study was comparable to that of children of the corresponding grade and sex in the national database of health check-ups in Japanese elementary school children [24].

### Definition of dietary supplement and assessment of dietary supplement use

Dietary supplements were defined as foods consumed orally as a capsule, tablet, powder, liquid, or other form to supplement the diet and contained one or more of the following ingredients: (i) vitamins, (ii) minerals, (iii) herbs or other botanicals, (iv) amino acids, and (v) ingredients of a concentrate, metabolite, constituent, or extract [13]. This definition of dietary supplements, as used in the USA, thus includes botanical supplements and bee products. In contrast, botanical supplements and bee products are considered “health foods” in Japan. Therefore, mothers were asked about whether their children used dietary supplements and related products that were covered by the definition of dietary supplements used in the USA. The researchers subsequently identified products that satisfied this definition.

Supplementary Figure 1 shows the questionnaire used in this study. Mothers were asked to respond to a question about whether their children used “dietary supplements,” “fortified foods,” or “health foods.” The question included “fortified foods” and “health foods” because these can meet the above criteria for dietary supplements. For example, while cod liver oil in the form of a gummy candy can be classified as a dietary supplement, mothers may consider it a fortified food. Additionally, while botanical or bee products can also be classified as dietary supplements, mothers may consider them health foods.

Mothers who responded that their children used dietary supplements were asked to indicate the brand names, manufacturers’ names, and, if available, to provide the barcodes on the labels of the dietary supplements. This information was subsequently used by nutrition researchers to identify the dietary supplements. Products that did not meet the criteria for the definition of dietary supplements were excluded. As a result, 442 products were identified as dietary supplements, 164 as fortified foods or naturally occurring foods, and three as over-the-counter drugs, while 61 could not be identified because of missing barcode or brand name data.

Types of dietary supplements were defined based on the ingredients contained in the dietary supplements, along with product-level information from labels. NVNM supplements were defined as dietary supplements that contained the following non-vitamin, non-mineral ingredients: (i) amino acids or protein; (ii) fatty acids or oils, such as eicosapentaenoic acid, docosahexaenoic acid, cod liver oil, and fish oil; (iii) botanicals (part of trees or plants, shrubs, and herbs or a component from a plant); (iv) probiotics or prebiotics; and (v) others [8, 25]. VM supplements were defined as dietary supplements containing any vitamins or minerals, but did not satisfy the definition for NVNM supplements [8]. Based on earlier studies, VM supplements were classified as follows: (i) multivitamin-minerals, (ii) multivitamins, (iii) multi-minerals, (iv) single vitamins, and (v) single minerals [26]. Multivitamins-minerals were defined as supplements containing three or more vitamins and at least one mineral. Multivitamins were defined as a single product containing two or more vitamins without any minerals. Multi-minerals were defined as a single product containing two or more minerals without any vitamins. Single vitamins and single minerals were defined as a single product containing one vitamin or mineral, respectively.

Mothers reported their child’s frequency of dietary supplement use by choosing from the following: less than once a week, once a week, 2–3 times per week, 4–6 times per week, or every day. Any use of dietary supplements was defined as dietary supplement use, regardless of frequency. Everyday use of dietary supplements was defined as dietary supplement use every day.

### Socio-demographic characteristics

Social and behavioral characteristics of dietary supplement users were assessed using questionnaires and included child school grade, sex, height, weight, health status, frequency of sports participation, household income, and maternal educational level [17, 18, 27]. Children’s body mass index (BMI) was categorized as underweight, normal-weight, or overweight using age- and sex-specific cut-off points defined by the International Obesity Task Force [28, 29]. Mothers reported their children’s general health status by choosing from the following five categories: poor, fair, good, very good, or excellent [18]. Because few mothers indicated that their children had poor health, the children’s general health status was subsequently grouped into the following four categories: poor or fair, good, very good, and excellent. Mothers reported their child’s frequency of sports participation by choosing from the following responses: never, once a week, 2–3 times per week, 4–6 times per week, or every day.

Mothers reported their annual household income (Japanese yen) by choosing from the following six categories: < 2,000,000, 2,000,000–3,999,999, 4,000,000–5,999,999, 6,000,000–7,999,999, 8,000,000–9,999,999, 10,000,000–11,999,999, and ≥ 12,000,000. Because few mothers indicated the highest household income category, household income was subsequently grouped into the following five categories: < 2,000,000, 2,000,000–3,999,999, 4,000,000–5,999,999, 6,000,000–7,999,999, 8,000,000–9,999,999, and ≥ 10,000,000. Mothers reported their educational level by choosing from the following responses: ≤ 12 years, 13–15 years, and ≥ 16 years.

### Statistical analyses

Multivariate logistic regression analyses were performed to estimate the odds ratio (OR) and 95% confidence interval (CI) of any use of dietary supplements to investigate the relationship between supplement use and the following demographic, social and behavioral characteristics: (i) child’s grade (reference: first grade); (ii) sex (reference: female); (iii) child’s BMI category (reference: normal-weight); (iv) health status (reference: poor or fair); (v) frequency of sports participation (reference: never); (vi) annual household income (reference: < 2,000,000 Japanese yen); and (vii) maternal educational level (reference: ≤ 12 years). All seven variates were simultaneously used for multivariate logistic regression models. No multicollinearity was observed.

We also examined the characteristics of children who used dietary supplements every day. Furthermore, we investigated the characteristics of users according to types of dietary supplements.

All statistical analyses were conducted using SAS version 9.4 (SAS Institute, Cary, NC, USA).

## Results

The mean (standard deviation) age of mothers and children was 39.8 (5.8) and 8.8 (1.7) years, respectively. Among the 4933 children who participated in this study, 333 (6.8%) were identified as dietary supplement users. Of users of any dietary supplements, 70 (21.0%) children used multiple dietary supplements and 117 (35.1%) children used dietary supplements every day.

Table 1 shows the characteristics of the study participants. Among all children, 2529 (51.3%) were male, and the majority of children had excellent or good health status. A total of 1706 (34.6%) mothers had the highest educational level (≥ 16 years).

**Table 1:** Characteristics of the study participants

	All children	
	n	(%)
Grade		
1	661	(13.4)
2	667	(13.5)
3	832	(16.9)
4	865	(17.5)
5	912	(18.5)
6	996	(20.2)
Sex		
Male	2529	(51.3)
Female	2404	(48.7)
BMI category^a^		
Underweight	713	(14.5)
Normal-weight	3119	(63.2)
Overweight	1101	(22.3)
Frequency of sports participation		
Never	1249	(25.3)
Once a week	841	(17.0)
2–3 times/week	1825	(37.0)
4–6 times/week	623	(12.6)
Every day	395	(8.0)
Reported health status		
Poor or fair	202	(4.1)
Good	1287	(26.1)
Very good	1360	(27.6)
Excellent	2084	(42.2)
Household income (10000 Japanese yen/year)		
< 200	488	(9.9)
200 to < 400	859	(17.4)
400 to < 600	1443	(29.3)
600 to < 800	1092	(22.1)
800 to < 1000	589	(11.9)
≥ 1000	462	(9.4)
Maternal educational level (years)		
≤ 12	1405	(28.5)
13 to 15	1822	(36.9)
≥ 16	1706	(34.6)

*BMI* body mass index

^a^BMI was categorized as underweight, normal-weight, or overweight based on age- and sex-specific cut-off points defined by the International Obesity Task Force [28, 29]

Table 2 shows dietary supplement use by type of dietary supplement. A total of 248 (5.0%) and 108 (2.2%) children used NVNM supplements and VM supplements, respectively. The most common type of dietary supplement was amino acids or protein (1.4%), followed by n–3 fatty acids or fish oil (1.1%), probiotics (1.0%), multivitamins (0.9%), botanicals (0.8%), and multivitamin-minerals (0.8%). Supplementary Table 1 lists the active ingredients of NVNM supplements. The most common active ingredient of botanical supplements was young barely leaf, while the most common active ingredient of other supplements was *Spirulina.*

**Table 2:** Types of dietary supplements used by children

	Number and percentage of supplement users^a^	
	n	(%)
Any type of dietary supplement	333	(6.8)
NVNM^b^	248	(5.0)
Amino acids or protein	68	(1.4)
n–3 fatty acids or fish oil	54	(1.0)
Botanicals	38	(0.8)
Probiotics	47	(1.0)
Others	43	(0.9)
VM^b^	108	(2.2)
Multivitamins-minerals	41	(0.8)
Multivitamins	42	(0.9)
Multi-minerals	16	(0.3)
Single vitamin^c^	10	(0.2)
Single mineral^d^	12	(0.2)

*NVNM* non-vitamin, non-mineral supplements, *VM* vitamin or mineral supplements

^a^Percentage of supplement users was calculated as follows: (users of each type of dietary supplement)/(total study participants [*n* = 4933])

^b^Total number of users of each type of non-vitamin, non-mineral supplement does not equal 248 because some children used more than one type of dietary supplement. Similarly, total number of users of each type of vitamin or mineral dietary supplement does not equal 108 because some children used more than one type of dietary supplement

^c^Single vitamins included vitamin B-group vitamins and vitamin C

^d^Single minerals included calcium and zinc

Table 3 shows the association between dietary supplement use and demographic, social, and health-related behavioral characteristics. Any dietary supplement use was significantly associated with the highest frequency of sports participation (OR, 2.58; 95% CI, 1.65–4.02), excellent health status (OR, 2.26; 95% CI, 1.35–3.76), highest household income (OR, 1.87; 95% CI, 1.13–3.10), highest maternal educational level (OR, 1.82; 95% CI, 1.31–2.52), and male sex (OR, 1.38; 95% CI, 1.09–1.75).

**Table 3:** Association between dietary supplement use and demographic, social, and health-related behavioral characteristics

	Any use of dietary supplements^a^				Everyday use of dietary supplements^b^			
	n^c^	OR	(95%CI)		n^c^	OR	(95%CI)	
Grade								
1	38	1.00	(reference)		16	1.00	(reference)	
2	32	0.88	(0.54,	1.43)	13	0.86	(0.41,	1.82)
3	55	1.13	(0.73,	1.77)	15	0.75	(0.36,	1.58)
4	53	1.04	(0.67,	1.61)	15	0.67	(0.33,	1.38)
5	72	1.28	(0.85,	1.94)	28	1.16	(0.62,	2.18)
6	83	1.38	(0.92,	2.07)	30	1.11	(0.59,	2.07)
Sex								
Male	201	1.38	(1.09,	1.75)	73	0.69	(0.47,	1.01)
Female	132	1.00	(reference)		44	1.00	(reference)	
BMI category^d^								
Underweight	38	0.75	(0.52,	1.07)	13	0.69	(0.38,	1.25)
Normal-weight	225	1.00	(reference)		83	1	(reference)	
Overweight	70	0.99	(0.73,	1.33)	21	0.84	(0.50,	1.40)
Frequency of sports participation								
Never	52	1.00	(reference)		21	1.00	(reference)	
Once a week	49	1.29	(0.86,	1.94)	12	0.75	(0.36,	1.54)
2–3 times/week	145	1.86	(1.33,	2.58)	47	1.46	(0.86,	2.47)
4–6 times/week	48	1.85	(1.22,	2.80)	23	2.19	(1.18,	4.05)
Every day	39	2.58	(1.65,	4.02)	14	2.12	(1.05,	4.29)
Health status								
Poor or fair	20	1.00	(reference)		42	1.00	(reference)	
Good	102	1.29	(0.97,	1.72)	31	1.16	(0.72,	1.87)
Very good	95	1.61	(1.21,	2.13)	35	1.52	(0.95,	2.41)
Excellent	116	2.26	(1.35,	3.76)	42	2.88	(1.35,	6.13)
Household income (10000Japanese yen/year)								
< 200	26	1.00	(reference)		9	1.00	(reference)	
200 to < 400	37	0.81	(0.48,	1.36)	9	0.57	(0.23,	1.46)
400 to < 600	87	1.06	(0.67,	1.67)	34	1.24	(0.59,	2.63)
600 to < 800	91	1.37	(0.86,	2.17)	30	1.36	(0.63,	2.94)
800 to < 1000	38	0.99	(0.59,	1.68)	11	0.87	(0.35,	2.15)
≥ 1000	54	1.87	(1.13,	3.10)	24	2.56	(1.14,	5.74)
Maternal educational level (years)								
≤ 12	61	1.00	(reference)		25	1.00	(reference)	
13 to 15	125	1.51	(1.10,	2.09)	40	1.13	(0.68,	1.90)
≥ 16	147	1.82	(1.31,	2.52)	52	1.44	(0.86,	2.41)

Multivariate logistic regression was adjusted for all other variables examined

*BMI* body mass index, *CI* confidence interval, *OR* odds ratio

^a^Any use of dietary supplements was defined as dietary supplement use, regardless of frequency (*n* = 333)

^b^Everyday use of dietary supplements was defined as dietary supplement use every day (*n* = 117)

^c^Number of dietary supplement users

^d^BMI was categorized as underweight, normal-weight, or overweight based on age- and sex-specific cut-off points defined by the International Obesity Task Force [28, 29]

Table 4 shows the association between dietary supplement use and demographic, social, and health-related behavioral characteristics by type of dietary supplement. Males were significantly more likely to use amino acids or protein than females (OR, 2.40, 1.38–4.18). There were no sex differences between users and non-users of other types of supplements. Compared to the lowest frequency of sports participation, the highest frequency of sports participation was significantly associated with higher odds of use of amino acids or protein (OR, 6.06; 95% CI, 1.78–20.6) and multivitamins (OR, 3.56; 95% CI, 1.11–11.5). The highest level of maternal education was significantly associated with higher odds of use of multivitamin-minerals (OR, 3.39; 95% CI, 1.20–9.58), compared to the lowest level of maternal education.

**Table 4:** Association between use of each type of dietary supplement and demographic, social, and health-related behavioral characteristics

	Amino acids or protein				n3-fatty acids				Botanicals				Probiotics				Multivitamins-minerals supplements				Multivitamins supplements			
	n	OR	(95%CI)		n	OR	(95%CI)		n	OR	(95%CI)		n	OR	(95%CI)		n	OR	(95%CI)		n	OR	(95%CI)	
Grade																								
1	5	1.00	(reference)		11	1.00	(reference)		3	1.00	(reference)		6	1.00	(reference)		4	1.00	(reference)		6	1.00	(reference)	
2	10	2.14	(0.72,	6.33)	3	0.28	(0.08,	1.01)	3	1.00	(0.20,	5.02)	7	1.24	(1.24,	0.41)	5	1.30	(0.34,	4.90)	5	0.87	(0.26,	2.89)
3	12	1.87	(0.65,	5.44)	10	0.66	(0.26,	1.66)	7	1.76	(0.43,	7.16)	8	1.14	(1.14,	0.37)	6	0.87	(0.23,	3.28)	6	0.84	(0.26,	2.76)
4	12	1.79	(0.62,	5.16)	9	0.60	(0.24,	1.46)	8	2.15	(0.56,	8.22)	7	0.87	(0.87,	0.29)	4	0.80	(0.20,	3.24)	9	1.11	(0.39,	3.16)
5	14	1.78	(0.63,	5.02)	11	0.64	(0.27,	1.50)	9	2.00	(0.53,	7.52)	9	0.95	(0.95,	0.33)	7	1.31	(0.38,	4.56)	9	0.87	(0.30,	2.56)
6	15	1.83	(0.65,	5.11)	10	0.53	(0.22,	1.28)	8	1.75	(0.46,	6.73)	10	1.02	(1.02,	0.36)	15	2.53	(0.83,	7.77)	7	0.69	(0.23,	2.09)
Sex																								
Male	50	2.40	(1.38,	4.18)	28	0.97	(0.56,	1.69)	21	1.17	(0.60,	2.25)	26	1.03	(0.57,	1.86)	20	0.93	(0.50,	1.75)	23	1.03	(0.55,	1.95)
Female	18	1.00	(reference)		26	1.00	(reference)		17	1.00	(reference)		21	1.00	(reference)		21	1.00	(reference)		19	1.00	(reference)	
BMI category^a^																								
Underweight	8	0.77	(0.36,	1.65)	7	0.88	(0.39,	2.02)	3	0.53	(0.16,	1.75)	6	0.81	(0.34,	1.96)	8	1.86	(0.80,	4.31)	3	0.42	(0.13,	1.38)
Normal-weight	48	1.00	(reference)		33	1.00	(reference)		26	1.00	(reference)		33	1.00	(reference)		19	1.00	(reference)		32	1.00	(reference)	
Overweight	12	0.87	(0.45,	1.69)	14	1.23	(0.61,	2.45)	9	1.00	(0.44,	2.28)	8	0.72	(0.31,	1.67)	14	2.47	(1.17,	5.21)	7	0.71	(0.29,	1.71)
Frequency of sports participation																								
Never	4	1.00	(reference)		10	1.00	(reference)		6	1.00	(reference)		6	1.00	(reference)		10	1.00	(reference)		6	1.00	(reference)	
Once a week	11	3.64	(1.15,	11.6)	12	1.66	(0.71,	3.90)	14	3.29	(1.24,	8.69)	10	2.12	(0.76,	5.92)	4	0.58	(0.18,	1.86)	5	1.10	(0.33,	3.65)
2–3 times/week	33	4.99	(1.75,	14.2)	19	1.29	(0.59,	2.82)	16	1.79	(0.69,	4.65)	23	2.44	(0.98,	6.08)	19	1.32	(0.61,	2.89)	17	1.86	(0.72,	4.79)
4–6 times/week	12	5.37	(1.70,	17.0)	7	1.52	(0.56,	4.09)	1	0.33	(0.04,	2.78)	6	2.16	(0.68,	6.86)	5	1.12	(0.37,	3.36)	8	2.42	(0.79,	7.43)
Every day	8	6.06	(1.78,	20.6)	6	2.29	(0.80,	6.51)	1	0.54	(0.06,	4.55)	2	1.23	(0.24,	6.26)	3	1.02	(0.27,	3.83)	6	3.56	(1.11,	11.5)
Health status																								
Poor or fair	19	1.00	(reference)		3	1.00	(reference)		3	1.00	(reference)		2	1.00	(reference)		5	1.00	(reference)		1	1.00	(reference)	
Good	19	1.71	(0.94,	3.08)	19	0.92	(0.27,	3.16)	15	2.38	(0.65,	8.70)	21	2.11	(0.46,	9.67)	11	1.43	(0.64,	3.16)	15	1.49	(0.67,	3.31)
Very good	24	1.58	(0.84,	2.96)	17	0.75	(0.21,	2.61)	8	1.87	(0.86,	4.05)	12	3.12	(1.51,	6.44)	12	1.43	(0.63,	3.24)	12	2.29	(1.06,	4.91)
Excellent	22	1.89	(0.55,	6.50)	15	0.42	(0.12,	1.48)	12	0.98	(0.40,	2.42)	12	1.49	(0.67,	3.36)	13	3.89	(1.33,	11.4)	14	1.13	(0.14,	8.90)
Household income (10000 Japanese yen/year)																								
< 200	6	1.00	(reference)		3	1.00	(reference)		6	1.00	(reference)		4	1.00	(reference)		3	1.00	(reference)		5	1.00	(reference)	
200 to < 400	7	0.68	(0.22,	2.03)	3	0.46	(0.11,	2.79)	3	0.28	(0.07,	1.14)	2	0.28	(0.05,	1.53)	8	1.56	(0.41,	5.95)	2	0.21	(0.04,	1.09)
400 to < 600	14	0.74	(0.28,	1.94)	18	1.19	(0.55,	6.50)	7	0.36	(0.12,	1.08)	10	0.73	(0.23,	2.36)	9	0.87	(0.23,	3.27)	11	0.66	(0.22,	1.93)
600 to < 800	21	1.33	(0.52,	3.40)	16	1.39	(0.62,	7.62)	13	0.86	(0.31,	2.34)	19	1.75	(0.58,	5.29)	8	0.96	(0.25,	3.73)	11	0.75	(0.25,	2.24)
800 to < 1000	7	0.77	(0.25,	2.37)	4	0.78	(0.22,	4.60)	2	0.25	(0.05,	1.26)	4	0.67	(0.16,	2.76)	6	1.25	(0.30,	5.20)	3	0.34	(0.08,	1.49)
≥ 1000	13	1.91	(0.69,	5.29)	10	2.19	(0.85,	12.2)	7	1.05	(0.33,	3.34)	8	1.73	(0.50,	5.98)	7	1.79	(0.44,	7.30)	10	1.24	(0.39,	3.98)
Maternal educational level (years)																								
≤ 12	15	1.00	(reference)		8	1.00	(reference)		7	1.00	(reference)		5	1.00	(reference)		5	1.00	(reference)		8	1.00	(reference)	
13 to 15	21	0.97	(0.49,	1.92)	26	0.93	(1.01,	5.06)	16	1.56	(0.63,	2.85)	22	2.85	(1.06,	7.62)	17	2.71	(0.98,	7.47)	9	0.83	(0.31,	2.18)
≥ 16	32	1.35	(0.69,	2.63)	20	0.70	(0.67,	3.76)	15	1.47	(0.56,	2.36)	20	2.36	(0.85,	6.54)	19	3.39	(1.20,	9.58)	25	2.21	(0.93,	5.25)

Multivariate logistic regression was adjusted for all other variables examined

Use of dietary supplements was defined as children who used dietary supplements, regardless of frequency

*BMI* body mass index, *CI* confidence interval, *OR* `odds ratio

^a^BMI was categorized as underweight, normal-weight, or overweight based on age- and sex-specific cut-off points defined by the International Obesity Task Force [28, 29]

## Discussion

We found that the most common type of dietary supplement was amino acids, followed by n–3 fatty acids or fish oil, and probiotics. Our findings showed that overall dietary supplement use was more prevalent in males, sports participants, and those with excellent health status, higher maternal educational level and household income. Furthermore, the characteristics of dietary supplement users differed by type of supplement.

A few studies have investigated NVNM supplement use among school-aged children [18, 20]. The National Health Interview Survey in the USA showed that the most common NVNM supplement among children and adolescents aged 4–17 years old was botanicals, followed by n–3 fatty acids and probiotics [20]. Furthermore, The National Health and Nutrition Examination Survey in the USA showed that children aged 0–19 years old used NVNM, including n–3 fatty acids and probiotics [18]. Overall, the types of NVNM used by children and adolescents in the present study, such as n–3 fatty acids, botanicals, and probiotics, were consistent with those reported in earlier studies [18, 20].

In the present study, amino acids and protein supplements were the most common dietary supplements among elementary school children. Similarly, earlier studies in Italy, Canada, and Australia showed that adolescents and young adults use amino acids and protein supplements [30–32]. Findings from the present study also showed that amino acid and protein supplement use was associated with sports participation. Consistent with this, a systematic review reported that gym users take amino acids or protein supplements, which have been shown to enhance muscle development, sports performance, and to help exercise recovery [33]. Children and their proxy may expect to enhance muscle development, sports performance by dietary supplements. However, earlier studies limited in adult studies [33]. It should be noted that data is lacking for efficacy and safety for dietary supplement use in children. Monitoring safety and confirm efficacy of dietary supplement use in children are needed.

We also found that sports participation was associated with use of multivitamin supplements, which is consistent with reports from a systematic review [34]. Sports participants may expect multivitamin supplements to improve performance. Guidelines for dietary supplement use for sports participants are also needed.

We found that the highest frequency of sports participation was associated with the highest odds of dietary supplement use in children, suggesting that children who are enthusiastic about sports use dietary supplements. Coaches of sports clubs may recommend that children use dietary supplements [35]. However, it is important for coaches and parents to understand current evidence on the efficacy and safety of dietary supplement use in children. Additional studies are needed to gain a more detailed understanding of dietary supplement use in sports club participants.

Supplements of n–3 fatty acids, fish oil, and cod liver oil were another common NVNM used by children in the present study. A systematic review reported that intake of n–3 fatty acids may improve cognitive performance in children and adolescents [36]. Cod liver oil is mostly used in coastal areas, including in the UK and northern Europe, where fish is readily available [37]. In Japan, elementary school children traditionally use cod liver oil at school to improve their nutrition status [38].

Botanical supplements are a major NVNM supplement used around the world [20, 30, 39, 40]. *Echinacea* is the most common botanical supplement in Western countries [20, 30, 39]. *Echinacea*, also known as coneflower, of family *Astraceae*, is endemic to North America and is a known immunostimulant and widely used as a dietary supplement in North America and Europe [41]. The present study showed that Japanese children also use *Echinacea*, although it was not the major botanical supplement. Rather, the most common botanical supplement was young barely leaf. Young barley leaf, also known as barley grass or *Hordeum vulgare L.*, is widely distributed and cultivated in East Asia. Young barley leaf is rich in dietary fiber, protein, fatty acids, and vitamins and minerals [42].

Other types of supplements in the present study included *Spirulina*, a microscopic filamentous alga [43]. A Korean study showed that *Spirulina* was a relatively common supplement among children [40]. In adults, *Spirulina* is known for its antioxidant, anti-aging and anti-cancer effects, and for enhancing immunity [44]. In terms of nutrition, *Spirulina* is rich in protein, essential fatty acids, vitamins, and minerals [43]. Mothers may expect *Spirulina* to promote growth in children.

Dietary supplements claim various positive effects. However, unlike medicines, dietary supplements do not require government certification of efficacy or safety before being sold on the market. Some systematic reviews have demonstrated the efficacy of dietary supplements used in this study [33, 36]. One review indicated that protein supplements may increase muscle mass during exercise training in adults [33]. Another reported that n–3 polyunsaturated fatty acid supplements may improve cognition in children, although the evidence varies among studies [36]. While dietary supplement use appears in general to be safe, harmful effects are sometimes reported. For example, a substantial number of botanical supplements are linked to adverse effects, including liver and kidney toxicity [45]. Although direct causal inference of these adverse effects has not been established, information on possible adverse effects should be provided to dietary supplement users and their parents.

In the present study, inquiry into the brands and manufacturers of the dietary supplements used by participants enabled investigation of newly sold supplements and supplements marketed to children. However, some limitations of this study should be acknowledged. First, study participants were limited to mothers who use the internet, because study participants were recruited through email and the website of a research agency. This may limit the generalizability of our findings. However, more than 90% of Japanese use the internet [46]. Furthermore, the characteristics of the study participants were similar to those in the national survey [21–24]. For example, household income among the study participants was similar to that among households with children in the national survey [22]. Second, barcodes or brand names of dietary supplements were not provided for a substantial number of products, which may have affected the results on the prevalence of use of each type of dietary supplement. These results should therefore be interpreted with caution. Third, the number of children using some types of dietary supplements was too small to detect meaningful differences in the characteristics between users and non-users.

In conclusion, this study revealed that Japanese children primarily use NVNM supplements. NVNM should thus be included in future studies aimed at monitoring dietary supplement use in addition to VM. We also found that dietary supplement use in children was associated with sports participation. Guidelines for dietary supplement use among children should target sports participants.

## Supplementary Information

**Additional file 1: Supplementary Table 1.** Active ingredients of NVNM supplements. **Supplementary Figure 1.** Questionnaire used in this study.

## Data Availability

The data sets generated and analyzed in the present study are not publicly available because we did not obtain informed consent from the participants for the open use of individual data.

## Abbreviations

BMI: Body mass index
CI: Confidence interval
NVNM: Non-vitamin, non-mineral
OR: Odds ratio
VM: Vitamin or mineral
