# Supplementary material for: Dietary supplement use in elementary school children: a Japanese web-based survey
Source: Environ Health Prev Med. 2021 Jun 5;26:63. doi: 10.1186/s12199-021-00985-7 (PMC8180069; doi:10.1186/s12199-021-00985-7)
Supplement: Supplementary file 1 — Additional file 1: Supplementary Table 1. Active ingredients of NVNM supplements. Supplementary Figure 1. Questionnaire used in this study. [file 12199_2021_985_MOESM1_ESM.doc]

# Supplementary Table 1. Active ingredients of NVNM supplements

|  | Number of NVNM users |
| --- | --- |
| Amino acids and protein |  |
| Arginine | 4 |
| Citrulline | 1 |
| Isoleucine | 1 |
| Leucine | 1 |
| Valine | 1 |
| Peptides from milk | 43 |
| Whey protein | 30 |
| n-3 fatty acids and fish oil |  |
| n-3 fatty acid | 31 |
| Fish oil | 23 |
| Botanicals |  |
| Young barely leaf | 23 |
| Blueberry | 7 |
| Kale | 6 |
| Chlorella | 5 |
| Euglena | 4 |
| Acerola | 3 |
| *Angelica keiskel* | 3 |
| Garlic | 2 |
| *Peucedanum japonicum* | 2 |
| Red clover | 2 |
| Aloe | 1 |
| *Echinacea* | 1 |
| Ginseng | 1 |
| Mulberry leaf | 1 |
| Soy products | 1 |
| Probiotics |  |
| Lactobacillus species | 46 |
| Bifidobacterium species | 2 |
| Yeast | 1 |
| Others |  |
| *Spirulina* | 19 |
| Collagen | 14 |
| Cod liver oil | 9 |
| Hyaluronic acid | 7 |
| Bee products | 5 |
| Euglena | 4 |
| Fiber | 3 |
| Alpha GPC | 2 |
| Coenzyme Q | 2 |
| Glucosamine | 2 |
| Lactoferrin | 2 |
| Lecithin | 2 |
| Oyster | 2 |
| Black vinegar | 1 |
| Carnitine | 1 |
| Ceramide | 1 |
| Chondroitin | 1 |
| Enzyme | 1 |
| Fulvic acid | 1 |
| Ornithine | 1 |
| Placenta | 1 |
| Proteoglycan | 1 |
| Softshell | 1 |

NVNM, non-vitamin, non-mineral supplements

# NVNM supplements sometimes included other active ingredients.

Supplementary Figure 1. Questionnaire used in this study

Child sex

○ Male

○ Female

Child birthday ⃞ year ⃞ month ⃞ day

Height ⃞ ⃞ ⃞ . ⃞ cm

Weight ⃞ ⃞ ⃞ . ⃞ kg

In the past month

Child participation in sports clubs and other intense physical activity

○ Every day

○ 4–6 times per week

○ 2–3 times per week

○ Once a week

○ Never

Child health status

○ Excellent

○ Very good

○ Good

○ Fair

○ Poor

**Gather all relevant products to answer the questions below about use of dietary supplements, health foods, and fortified foods.**

Does your child use dietary supplements, health foods, or fortified foods? If so, how many types of these products does he/she use?

○ None

○ One

○ Two

○ Three

○ Four

○ Five or more

Does the label of these products have a barcode?

○ Yes

○ No

Please provide the product’s barcode number

Please provide the name of the company that produces the product

Please provide the name of the product

How frequently does your child use the product?

○ Less than once a week

○ Once a week

○ 2–3 times per week

○ 4–6 times per week

○ Every day.

How much of the product does your child use per day?

Amount ( ) Unit ( )

Please choose your most recent academic history.

○ Junior high school

○ High school

○ Technical school, vocational school, or junior college

○ University or graduate school

Please choose annual household income.

○ <2,000,000 Japanese yen

○ 2,000,000–3,999,999 Japanese yen

○ 4,000,000–5,999,999 Japanese yen

○ 6,000,000–7,999,999 Japanese yen

○ 8,000,000–9,999,999 Japanese yen

○ 10,000,000–11,999,999 Japanese yen

○ ≥12,000,000 Japanese yen
